# Five Decades of Research Progress in Air Pollution, Children’s Respiratory Health, and Emergency Department Visits: A Bibliometric Analysis

**DOI:** 10.7759/cureus.37151

**Published:** 2023-04-05

**Authors:** Afiqah Syamimi Masrani, Nik Rosmawati Nik Husain, Kamarul Imran Musa

**Affiliations:** 1 Department of Community Medicine, School of Medical Sciences, Universiti Sains Malaysia, Kota Bharu, MYS

**Keywords:** child and adolescent, bibliometric analyses, emergency department visit rates, respiratory disease, air pollution

## Abstract

Air pollution is a global environmental health concern. Our study aims to examine the collective scientific impact of air pollution, children's respiratory health, and emergency department visits during the last five decades. Original articles, review papers, and conference proceedings in the English language published from 1972 to 2022 were obtained after a comprehensive search of the Scopus database using the terms air pollution, children, respiratory health, and emergency department visit. The Biblioshiny web application of the R software (R Foundation for Statistical Computing, Vienna, Austria) was used to analyse the publication trend and identify the top authors and journals of the subject. The countries’ collaborative network was mapped, and the authors’ trending keywords were tracked using a thematic map. In total, 1,309 publications authored by 6,342 authors from 483 sources were retrieved. Three distinctive collaborative network clusters were observed, with the United States as the connecting central node. Among the 39 trending keywords identified, particulate matter had constantly been a motor theme with an emerging interest in individual pollutants, specific diseases, and time series analysis. In conclusion, political will is a strong driver for research on air pollution, children’s respiratory health, and emergency department visits, which is further enhanced by technological advancement that increases the availability and accessibility of air pollution and patient data. The trend for future studies is time series analysis and research on the impact of individual air pollutants on specific respiratory disorders in children.

## Introduction and background

The adverse effects of air pollution on human health were brought to light during the time of Hippocrates around 400 BC. In 1958, the World Health Organization released its first publication on the Air Pollution technical report series. The report was the first to evaluate the need for an air quality standard, but it was decided that there was not enough evidence to allow for establishing regulations intended to protect public health at that time. Currently, more than five decades after the report, air pollution is considered the single major environmental risk to human health worldwide, warranting three indicators in the global Sustainable Development Goals. More than 91% of the world’s population has been exposed to air pollution levels over WHO guideline limits as a result of rising hydrocarbon combustion product emissions that increased greenhouse gas emissions and caused global warming [1]. In 2016, air pollution was attributed to over 4.2 million premature deaths worldwide, of which nearly 300,000 were children under the age of five years [2].

Compared to adults, children are more likely to have adverse health impacts from exposure to air pollution due to a combination of behavioural, environmental, and physiological variables [2]. A significant burden of mortality and morbidity resulted from this greater susceptibility as children are exposed to indoor and ambient air pollution. There is compelling evidence from numerous studies that exposure to air pollution can raise the risk of malignancies and possibly even increase childhood obesity [3,4]. Specific to children’s respiratory health, air pollution has been shown to cause a decline in lung function as well as an increase in the risk of asthma, upper respiratory tract infection, pneumonia, and even death [5-7]. Even though these studies supported the harmful effects of air pollution on children’s health, the majority of the literature in this field concentrates on certain policies, interventions, and diagnoses [8].

The emergency department (ED) is the point of contact for the community for emergency health services. Nearly 20% of all children under 18 years old visit the ED annually, with approximately 25% presenting to the ED twice or three times [9,10]. Studies on the association of air pollution with pediatric ED visits have remained contradictory in the literature, with a positive association being more pronounced for conditions involving the respiratory system. For instance, a study in China found that a 10 μg/m^3^ increase in particulate matter with an aerodynamic diameter ≦ 10 µm (PM_10_) was associated with an inverse association with pediatric asthma visits to the ED (excess relative risks (ERR) -1.1, 95%CI: -2.06, -0.13) at lag 0 day [11], whereas another study in Taiwan found that PM_10_ was positively associated with pediatric asthma visit to the ED (adjusted relative risks (RR) 1.20, 95%CI: 1.13, 1.27) [12].

Air pollution, children’s respiratory health, and ED visits have all been the subject of extensive prior research. However, there hasn’t been any study of how these three distinct topics have been investigated collectively. Thus, as it is at the centre of scientific evaluation, it is necessary to measure the development, progress, and impact of global research on these combined issues. One method of evaluation is by thoroughly researching the literature that describes advancement in this area, known as bibliometric analysis. Bibliometric analysis is an effective method that can provide quantitative data on particular topics [13]. Furthermore, the identification of prominent authors and journals, the potential countries for collaboration, and the trending keywords on the subject provide relevant references to researchers, professionals, and others working on air pollution research as tools to address current problems such as transboundary air pollution and limitation in research. Hence, in this study, we aim to analyze the trend and research progress on air pollution, children’s respiratory health, and ED visit in the past five decades. 

This article was previously presented as a meeting abstract at the 3rd World Conference on Public Health on December 9, 2022.

## Review

Materials and methods

Data Source and Eligibility Criteria

The Scopus database was selected for data extraction as it has a wider range of scholarly information, with more than 40,000 indexed journals across all scientific fields. Only original articles, reviews, and conference proceedings in the English language published from 1972 to 2022 were considered eligible. Other publications, such as letters or editorials, were excluded.

Search Strategy

**Figure 1:**
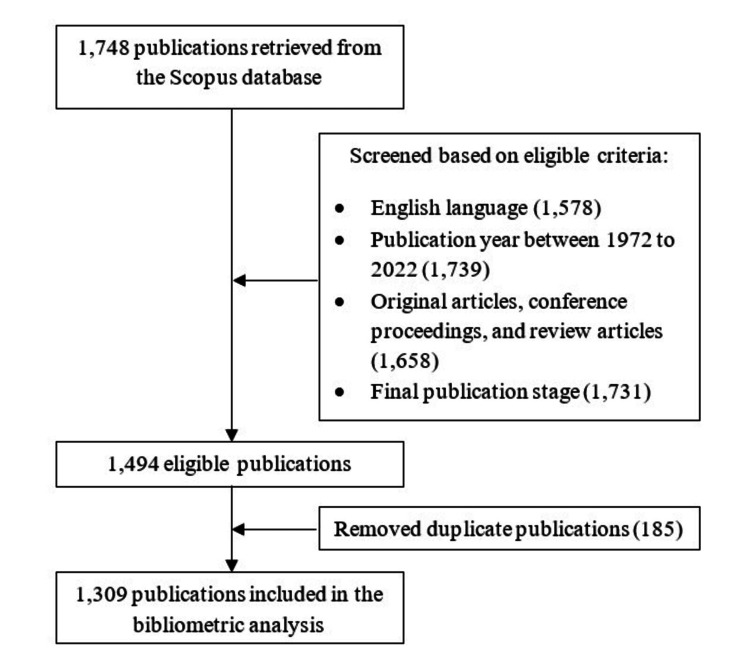
The flowchart of the research to search papers in databases

A comprehensive online search of the Scopus database was done on October 34, 2022. In the advanced search option of the Scopus database, using an appropriate combination of Boolean search operators, a total of four keywords and their medical subject headings (MeSH) terms were searched within the article title, abstract, and keywords: air pollution, children, respiratory health, and ED visit. However, considering that air pollution is a collective term used for contaminants in the air, we noted previous studies involving single pollutants only. Therefore, the main air pollutants namely particulate matter, carbon monoxide, nitrogen dioxide, sulphur dioxide, and ozone, were also included in the search query provided that the study was in the context of air pollution. Figure 1 depicts the flowchart of our research. A detailed search strategy is presented in Appendix 1.

Bibliometric Analyses

The metadata consisting of all the main information and characteristics of the included studies were retrieved and downloaded in the BibTex format. Data management and bibliometric analyses were conducted using the Bibliometrix package (version 4.0.1; K-Synth Srl, Naples, Italy) and Biblioshiny (K-Synth Srl) web apps under the R language (version 4.2.1) inside RStudio integrated development environment for R (2022.7.1.554; RStudio, Boston, Massachusetts, United States). The publication trend was constructed over 50 years. We identified the top 10 most relevant authors and the top 10 most relevant sources based on the total number of publications between 1972 and 2022. The top 10 sources' impact was then tabulated to compare the total number of citations and H-indexed citations. The publication by countries was mapped and a collaboration network among the top 20 countries with the highest publications on the topic from 1972 to 2022 was constructed. The frequency of the authors’ keywords was plotted against the years to demonstrate the trending topics in the given period. Finally, the ‘Thematic Map’ was developed based on four themes, including (i) motor themes, (ii) niche themes, (iii) emerging or declining themes, and (iv) transversal and basic themes. The map was constructed using a co-occurrence network to identify clusters of keyword plus, which are keywords assigned by journals after extracting words and phrases from the titles of cited articles.

Results

**Figure 2:**
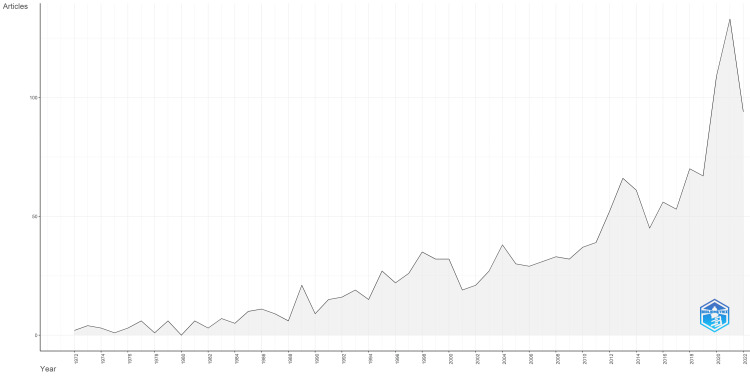
Annual scientific publications on air pollution, children’s respiratory health, and emergency department visits from 1972 to 2022

A total of 1,309 publications from 483 sources were retrieved within the 50 years between 1972 to 2022. Out of these, most are original articles (86.8%) followed by review papers (10.9%), and conference proceedings (2.3%); 37.1% of the research was funded. The topic has experienced an 8.1% annual growth rate (Figure 2), with an average of 47.1 citations per publication.

Authors and Journals

A total of 6,342 authors have contributed to the topic. Most publications (1,230; 94.0%) were co-authored with an average of 6.2 authors per publication. The top 10 most productive authors based on their total number of publications over the period of 50 years are depicted in Table 1. Author Bert Brunekeef has the highest number of published papers (20, 1.52%) followed by Gerard Hoek (17, 1.30%). Although author Kirk R. Smith has the highest total citation (3,700 citations) followed by Joel Schwartz (3,127 citations), author Bert Brunekreef has a higher author impact with 19 H-indexed publications followed by Gerard Hoek (15 H-indexed publications), and Joel Schwartz (14 H-indexed publications).

**Table 1:** Top 10 authors on air pollution, children's respiratory health and emergency department visits over five decades

Rank	Author	Field of interest	Institution	Country	Total number of publications	H-indexed publications	G-indexed publications	M-indexed publications	Total number of citations	Start of publication year
1	Bert Brunekreef	Environmental epidemiology	Utrecht University	Netherlands	26	19	26	0.559	1516	1990
2	Gerard Hoek	Environmental epidemiology	Utrecht University	Netherlands	24	15	24	0.441	1144	1990
3	Joel Schwartz	Environmental epidemiology	Harvard T.H. Chan School of Public Health	USA	16	14	16	0.4	3127	1989
4	Haidong Kan	Environmental health sciences	Fudan University	China	14	13	14	0.65	2142	2004
5	Kirk R. Smith	Global environmental health	University of California	USA	17	13	17	0.371	3700	1989
6	Yang Liu	Environmental health	Emory University	USA	20	12	20	0.343	584	1989
7	Isabelle Romieu	Environmental health	National Institute of Public Health	Mexico	12	12	12	0.375	2643	1992
8	Richard T. Burnett	Environmental epidemiology	University of Ottawa	Canada	11	11	11	0.282	2227	1985
9	Nigel Bruce	Public health	University of Liverpool	UK	10	10	10	0.385	2820	1998
10	Yanwu Zhang	Epidemiology	Anhui Medical University	China	17	10	17	0.556	584	2006

The top 10 most relevant journals on air pollution, children’s respiratory health, and ED visit are listed in Table 2 including the total number of articles published, total number of citations, and citation impacts of the articles published. Seven out of the 10 journals are focused on environmental sciences. The Environmental Research journal has been the top source on this topic since 1972 with a total of 59 articles published. Comparatively, the Environmental Health Perspectives journal has the highest total citation and H-indexed citation since 1975 with a total of 53 publications. Despite only starting to publish articles on the topic in the past decade, the Environmental Science and Pollution Research journal has ranked the 10th highest in the number of publications (18 articles) as opposed to the other journals of its time which published between one to two articles on the topic in the same period.

**Table 2:** Top 10 journals with the highest number of publications, journal impact, and the start of publication year on the topic

Rank	Journal	Total number of publications	H-indexed publications	G-indexed publications	M-indexed publications	Total citation	Start of publication year
1	Environmental Research	59	33	52	0.647	2,787	1972
2	Environmental Health Perspectives	53	38	53	0.792	5,882	1975
3	International Journal of Environmental Research and Public Health	46	17	28	1.000	813	2006
4	Environment International	29	21	29	0.525	1,191	1983
5	Environmental Health: A Global Access Science Source	26	19	26	1.188	1,017	2007
6	Science of the Total Environment	25	16	25	0.842	1,028	2004
7	American Journal of Respiratory and Critical Care Medicine	23	23	23	0.793	3,781	1994
8	American Journal of Epidemiology	22	19	22	0.432	2,089	1979
9	Pediatric Pulmonology	20	9	21	0.237	672	1985
10	Environmental Science and Pollution Research	18	10	17	0.769	294	2010

Countries and Collaboration Network

Overall, 72 countries have contributed to the production of publications on the topic of air pollution, children’s respiratory health, and ED visits. The top global manufacturing countries are the leading frontiers in this topic with the United States of America (USA) having the highest number of publications produced (407 publications) followed by China (122 publications), and the United Kingdom (68 publications). Meanwhile, the United Kingdom leads in the number of publications with international collaboration compared to their overall publications, as evidenced by the single to multiple-country production of 0.353 followed by Canada (0.345) and China (0.262). Table 3 summarizes the top 10 countries along with their total number of publications, annual publishing frequency, single-country publications, multi-country publications, and the proportion of single-country publications to multi-country publications.

**Table 3:** Top 10 countries producing publications relating to air pollution, children’s respiratory health, and emergency department visit SCP: single country production; MCP: multiple country production

Rank	Country	Number of publications	Frequency	SCP	MCP	MCP_SCP ratio
1	USA	407	0.341	339	68	0.167
2	China	122	0.102	90	32	0.262
3	United Kingdom	68	0.057	44	24	0.353
4	Canada	58	0.049	38	20	0.345
5	Italy	48	0.040	40	8	0.167
6	India	37	0.031	32	5	0.135
7	Australia	36	0.030	27	9	0.250
8	Japan	28	0.024	22	6	0.214
9	Brazil	24	0.020	19	5	0.208
10	France	24	0.020	23	1	0.042

**Figure 3:**
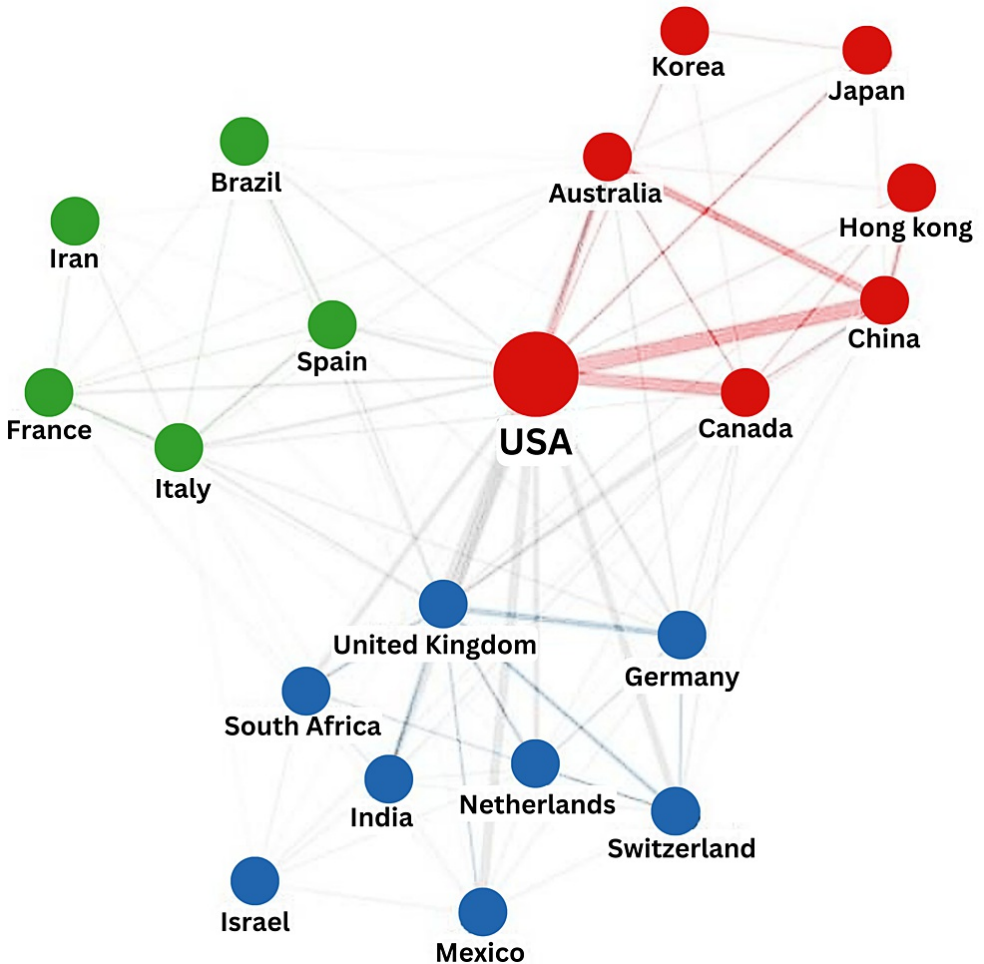
Collaborative network of countries publishing research on air pollution, children’s health, and emergency department visits USA: United States of America

Three distinctive clusters of collaborative networks are observed within the top 20 countries (Figure 3). Geographically, these clusters can be divided into the East Asia cluster, Southwest Europe cluster, and Central-Northwestern Europe cluster. The USA acts as the centralizing node that connects these three clusters with the United Kingdom and Italy being the collaborative node for their respective clusters.

Trending Keyword and Thematic Map

**Figure 4:**
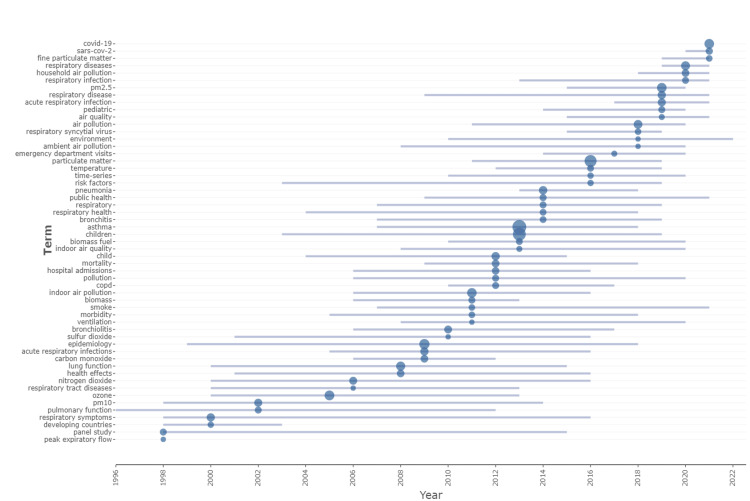
Trending topics and keywords using keywords plus for air pollution, children’s respiratory health, and emergency department visit from 1972 to 2022 The line indicates the occurrence of the keyword; the size of the bubble indicates the frequency of the keyword being used within the year COVID-19: coronavirus disease 2019; SARS-CoV-2: severe acute respiratory syndrome coronavirus 2; PM: particulate matter

Over the period of five decades, 39 trending author keywords were identified (Figure 4). The trending keywords have a minimum word frequency of 200 and have been featured at least five times per year in the publications included between 1972 and 2022. We observe four trends in the trending keywords. The first is the change from panel studies, which were gradually replaced by time series studies between 2010 to 2015. Second, in terms of children’s respiratory health, we can see that the trend shifted from general symptoms to the pathophysiology of a disease, to specific diseases such as bronchiolitis, asthma, and the most recent coronavirus disease 2019 (COVID-19). Third, we observed changes in the type of air pollutant being studied. Among the air pollutants, PM_10_ was the first to start trending from 1998 up to 2014 when a shift to particulate matter less than 2.5 microns (PM_2.5_) and subsequently fine particulate matter in 2018 was observed. Finally, we observed that the population sample of the research had changed from hospital admissions and mortality in 2009 to ED visits in 2014.

**Figure 5:**
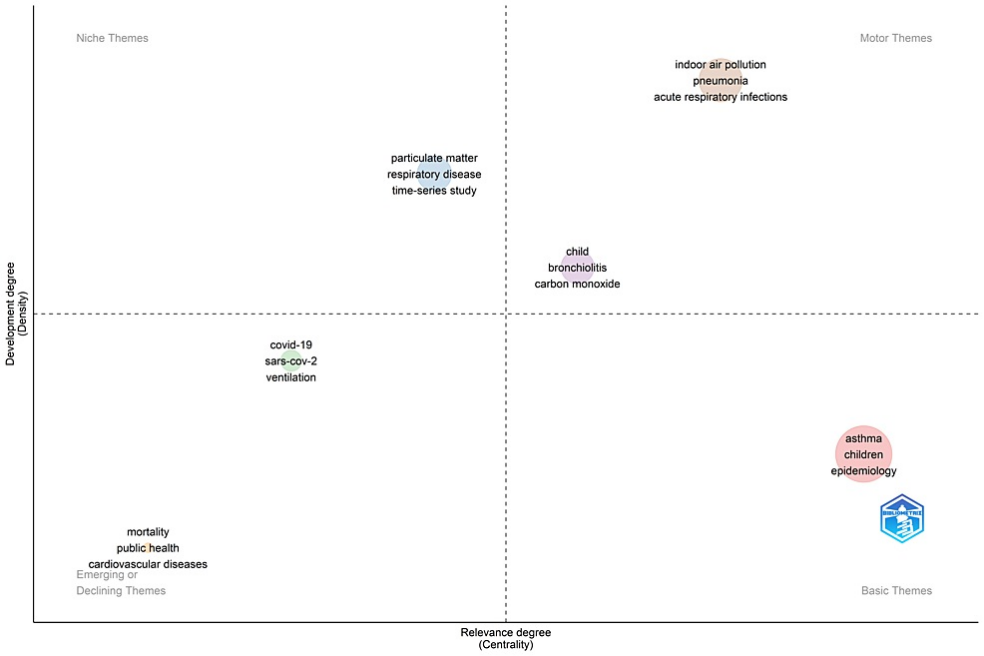
Thematic map of authors’ keywords on the topic of air pollution, children’s respiratory health, and emergency department visits COVID-19: coronavirus disease 2019; SARS-CoV-2: severe acute respiratory syndrome coronavirus 2

Based on Callon’s centrality (x-axis) and density (y-axis) rank values, the keyword clusters are shown as bubbles on the thematic map. The frequency of each word in the cluster is reflected in the size of the bubble. The level of interaction between a network cluster and other clusters visible in the same graph is known as centrality. It can be viewed as a gauge of a theme’s relevance in the growth of the research field. The internal strength of a cluster network is measured by density, which can be interpreted as a gauge of theme development. The three keywords air pollution, children, and respiratory diseases are observed as motor themes with varying relevance and development degree (Figure 5). Specific respiratory diseases such as pneumonia and bronchiolitis have a high degree of development, whilst COVID-19 has become an emerging theme.

Discussion

Prior to the 1980s, minimal fluctuation in the trend of publications on air pollution, children's respiratory health, and ED visits was observed, with less than 10 papers each year. After the United Nations Economic Commission for Europe (UNECE) adopted the Helsinki Protocol on Further Reduction of Sulphur Emissions in 1985, we observed an upsurge in the velocity of study on this topic. The 25 participating nations from Europe and North America agreed to reduce their yearly sulphur emissions or transboundary fluxes by at least 30% by 1993 while also conducting national research to determine whether further reductions are necessary [14]. The Gothenburg Protocol was also formed by UNECE in 1999 to address pollutants that contribute to acidification and ground-level ozone, which led to an increase in the number of publications published on the subject between 1999 to 2001 [14]. The Clean Air Act of 1970 and its numerous revisions, including the Clear Skies legislation in 2003 to establish the National Ambient Air Quality Standards for regulating emissions of dangerous air pollutants, stimulated research at the same time in the USA. In order to minimise greenhouse gas emissions, $27 billion in subsidies would be given to green banks nationwide under the most recent update to the act, which President Joe Biden signed in 2022 [15]. As a result, we predict that research into greenhouse gas reduction will grow.

Spikes in publications were observed to be triggered not only by legislation changes but also by the identification of new evidence on sources of air pollutants or their environmental impact. In the 1980s, for example, acid deposition caused by pollutants emitted during fossil fuel combustion resulted in the decline of forest health in Europe and North America. In 1995, the first satellite for passive remote sensing atmospheric composition, Global Ozone Monitoring Experiment (GOME), was launched, thus improving data on global ozone [14]. Subsequently, in the early 21st century, China and other Asian countries gradually replaced Europe as the major pollutant emissions leading to the Beijing fog in 2012, which was as detrimental as the great smog in London in the 1950s [14]. As a result, more air quality monitoring stations were established worldwide, leading to increased data and subsequent research. But then, the COVID-19 pandemic occurred in 2020, and a decline in trend was observed. However, we expect this decline to be temporary as new research was brought forth by the dramatic reduction in transport-related emissions of sulphur dioxide, nitrogen dioxides, volatile organic compounds, and particulate matter globally [16-19].

All five prominent authors on the topic of air pollution, children’s respiratory health, and ED visits before the 21st century are from Europe, specifically the Netherlands, and the USA. Even Dr. Yi Liu, presently employed at Tsinghua University in Taiwan, was once a member of the Group of Environmental Policy at Wageningen University in the Netherlands. They mostly majored in environmental sciences and trained in epidemiology and public health. The start of the 21st century shows a rise in authors with mostly engineering backgrounds from Asian countries. The shift in the background of the prominent authors is a testament to the diverse areas of environmental health that embraces advancement in technology and the geographical spread of the concern and source of air pollution.

The rising trend of international co-authorships reflects an evolving international collaboration network in research. Collaborations based on geographical proximity allow comparison of relatively similar environments controlled for seasonal and demographic differences. In addition to the location of the countries, we postulate that the separation of the European countries into two clusters may be due to economic or political reasons. For example, countries in the blue cluster are trading partners with the United Kingdom, while countries in the green cluster are semi-periphery countries. These clusters share similar manufacturing and exportation landscapes, which may produce homogenous sources of air pollution [20]. Contrarily, the clustering of the Asian countries with the USA may be due to the Asia-Pacific Network for Global Change Research (APN) that was called into cooperation by the USA in 1990. Since then, APN funding has supported networking and capacity building between research groups in Asia. One such project is the collaboration among scientists from Australia, China, India, Italy, Japan, the Republic of Korea, and the USA that compared regional climate projections as part of the assessment reports of the Intergovernmental Panel on Climate Change (IPCC) [21].

The leading countries in air pollution research also happen to be the top manufacturing nations in the world. Interestingly, these countries, aside from India, are not the most polluted. In fact, they range between rank 22nd to 109th, leading us to two possible reasons: industry-driven research and transboundary air pollution. Europe and North America had dominated the emissions and suffered the majority of adverse health effects until the latter decades of the 20th century [14]. To mitigate this increasing concern, industry-driven research was in full force for evidence on ways to control emission and their effectiveness. In the early 21st century, manufacturing in East and South Asia had risen dramatically. This put the primary environmental and political air quality issues (acid rain, forest degradation, and ground-level ozone) in the spotlight.

Over time, we saw variations in the study design choices, population samples, examined exposures (air pollutants), and health outcomes (respiratory disorders). The changing preference for time series studies and specific patient diagnosis shows a rising interest in the effects of long-term and short-term exposure to air pollution on children’s respiratory health. We believe that easier access to quality patients and air pollution data, as well as the availability of analysis software, made this possible. Changes in the pollutant being investigated may be a sign of advancements in air quality monitoring technologies. Therefore, if the technology generally becomes available in a few years, ultrafine particle pollution might also be trending. Likewise, the technology used in the medical industry is revolutionary for the advancement of research. For instance, researchers now have access to a wider range of patient data due to the ED’s use of the electronic medical records system.

Within two years of the COVID-19 pandemic, the term "COVID-19" appeared in the category of emerging themes. The speed with which publications on this topic were published showed how hard journals had worked throughout time to reduce the financial barrier to publication and enable quick information dissemination. In the same cluster, the term "ventilator" also appeared. The leading eigenvalue-clustering algorithm was used to plot the keywords in the theme map. This algorithm determines how central a keyword is based on how closely related the words are and how significant their neighbours are [22]. Consequently, it highlights the importance of research into disease management.

In our study, the main limitation was during data collection. As air pollution is a global issue, we know that much other research wasn’t written in the English language, which may produce a lack of representation of the actual publications. In addition, some research might not get published for various reasons, including financial limitations for the publication fees, and even if they were published, they might not be published in journals indexed by Scopus. However, the Scopus database is one of the main bibliographic databases compatible with the bibliometric analysis software. Therefore, future studies may complement our findings by comparing them to data from publications or databases with a more varied language.

## Conclusions

Our investigation has led to three main findings. First and foremost, political will and global movements are powerful research proponents, particularly because air pollution knows no borders. Second, as technology develops, data accessibility and availability both increase. This enables academics to investigate specific areas of the current topic. Not to mention the progress of analyzing software. Finally, the trend for future study is time series analysis, which monitors the impact of individual pollutants on certain respiratory disorders in children.

## Appendices

Appendix 1

Search Strategy

TITLE-ABS-KEY(("air pollution" OR "ambient" OR "carbon monoxide" OR "nitrogen dioxide" OR "ozone" OR "particulate matter" OR "sulphur dioxide" OR "sulfur dioxide" OR "air quality") AND ("child" OR "children" OR "paediatric" OR "pediatric" OR "infant" OR "school") AND ("respiratory" OR "lung" OR "pulmonary") AND ("emergency department" OR "emergency room" OR "emergency visit" OR "outpatient" OR "acute")) AND ( LIMIT-TO ( PUBYEAR,2022) OR LIMIT-TO ( PUBYEAR,2021) OR LIMIT-TO ( PUBYEAR,2020) OR LIMIT-TO ( PUBYEAR,2019) OR LIMIT-TO ( PUBYEAR,2018) OR LIMIT-TO ( PUBYEAR,2017) OR LIMIT-TO ( PUBYEAR,2016) OR LIMIT-TO ( PUBYEAR,2015) OR LIMIT-TO ( PUBYEAR,2014) OR LIMIT-TO ( PUBYEAR,2013) OR LIMIT-TO ( PUBYEAR,2012) OR LIMIT-TO ( PUBYEAR,2011) OR LIMIT-TO ( PUBYEAR,2010) OR LIMIT-TO ( PUBYEAR,2009) OR LIMIT-TO ( PUBYEAR,2008) OR LIMIT-TO ( PUBYEAR,2007) OR LIMIT-TO ( PUBYEAR,2006) OR LIMIT-TO ( PUBYEAR,2005) OR LIMIT-TO ( PUBYEAR,2004) OR LIMIT-TO ( PUBYEAR,2003) OR LIMIT-TO ( PUBYEAR,2002) OR LIMIT-TO ( PUBYEAR,2001) OR LIMIT-TO ( PUBYEAR,2000) OR LIMIT-TO ( PUBYEAR,1999) OR LIMIT-TO ( PUBYEAR,1998) OR LIMIT-TO ( PUBYEAR,1997) OR LIMIT-TO ( PUBYEAR,1996) OR LIMIT-TO ( PUBYEAR,1995) OR LIMIT-TO ( PUBYEAR,1994) OR LIMIT-TO ( PUBYEAR,1993) OR LIMIT-TO ( PUBYEAR,1992) OR LIMIT-TO ( PUBYEAR,1991) OR LIMIT-TO ( PUBYEAR,1990) OR LIMIT-TO ( PUBYEAR,1989) OR LIMIT-TO ( PUBYEAR,1988) OR LIMIT-TO ( PUBYEAR,1987) OR LIMIT-TO ( PUBYEAR,1986) OR LIMIT-TO ( PUBYEAR,1985) OR LIMIT-TO ( PUBYEAR,1984) OR LIMIT-TO ( PUBYEAR,1983) OR LIMIT-TO ( PUBYEAR,1982) OR LIMIT-TO ( PUBYEAR,1981) OR LIMIT-TO ( PUBYEAR,1979) OR LIMIT-TO ( PUBYEAR,1978) OR LIMIT-TO ( PUBYEAR,1977) OR LIMIT-TO ( PUBYEAR,1976) OR LIMIT-TO ( PUBYEAR,1975) OR LIMIT-TO ( PUBYEAR,1974) OR LIMIT-TO ( PUBYEAR,1973) OR LIMIT-TO ( PUBYEAR,1972) OR LIMIT-TO ( PUBYEAR,1971) OR LIMIT-TO ( PUBYEAR,1970) ) AND ( LIMIT-TO ( LANGUAGE,"English" ) ) AND ( LIMIT-TO ( DOCTYPE,"ar" ) OR LIMIT-TO ( DOCTYPE,"re" ) OR LIMIT-TO ( DOCTYPE,"cp" ) ) AND ( LIMIT-TO ( PUBSTAGE,"final" ) )
